# RETRACTION: Effect of Bevacizumab in Combination With Chemotherapy for Ovarian Cancer on Wound Healing in Patients: A Meta‐Analysis

**DOI:** 10.1111/iwj.70358

**Published:** 2025-03-18

## Abstract

**RETRACTION**: XueY. and ZhaoF. , “Effect of Bevacizumab in Combination with Chemotherapy for Ovarian Cancer on Wound Healing in Patients: A Meta‐Analysis,” International Wound Journal 21, no. 4 (2024): e14531, 10.1111/iwj.14531.38151891 PMC10961034

The above article, published online on 27 December 2023, in Wiley Online Library (http://onlinelibrary.wiley.com/), has been retracted by agreement between the journal Editor in Chief, Professor Keith Harding; and John Wiley & Sons Ltd. Following an investigation by the publisher, all parties have concluded that this article was accepted solely on the basis of a compromised peer review process. The editors have therefore decided to retract the article. The authors did not respond to our notice regarding the retraction.

**RETRACTION**: Y. Xue and F. Zhao , “Effect of Bevacizumab in Combination with Chemotherapy for Ovarian Cancer on Wound Healing in Patients: A Meta‐Analysis,” International Wound Journal 21, no. 4 (2024): e14531, 10.1111/iwj.14531.38151891 PMC10961034

The above article, published online on 27 December 2023, in Wiley Online Library (http://onlinelibrary.wiley.com/), has been retracted by agreement between the journal Editor in Chief, Professor Keith Harding; and John Wiley & Sons Ltd. Following an investigation by the publisher, all parties have concluded that this article was accepted solely on the basis of a compromised peer review process. The editors have therefore decided to retract the article. The authors did not respond to our notice regarding the retraction.

